# Postcardiotomy Mechanical Circulatory Support in Two Infants with Williams' Syndrome

**DOI:** 10.1155/2014/795726

**Published:** 2014-03-11

**Authors:** Constantinos A. Contrafouris, Andrew C. Chatzis, Meletios A. Kanakis, Prodromos A. Azariadis, Fotios A. Mitropoulos

**Affiliations:** Department of Paediatric and Congenital Heart Surgery, Onassis Cardiac Surgery Centre, 356 Syngrou Avenue, Kallithea, 17674 Athens, Greece

## Abstract

Supravalvar aortic stenosis (SVAS) in patients with Williams' syndrome is often accompanied by coronary, pulmonary, and even myocardial lesions and therefore associated with increased perioperative morbidity and mortality. Extracorporeal membrane oxygenation (ECMO) provides reliable short-term mechanical circulatory support to patients, especially young, in acute postoperative cardiac failure when conventional means are ineffective. The incorporation of centrifugal pumps in these systems has made their use more efficient and less traumatic. We describe our experience of using the Levitronix CentriMag pump in two patients with Williams' syndrome who underwent surgical correction of supravalvular aortic stenosis.

## 1. Introduction

Supravalvar aortic stenosis (SVAS) is commonly found in patients with Williams' syndrome. The association of SVAS and pulmonary artery stenosis with mental retardation and distinctive facial features is known as the Williams-Beuren syndrome [1, 2]. Prominent feature is the diseased media of the ascending aorta, which also may affect the coronary arteries as well as the pulmonary vasculature, causing stenoses. Although there is supporting evidence of a genetic defect in elastin, it is also true that the disease affects all layers of the major vessels along with other parts of the cardiovascular system, including the cardiac valves, the myocardium, and the peripheral vessels [3–5].

Although low cardiac output is not uncommon following surgery for congenital heart disease in the paediatric population, conventional methods of treatment prove that efficient and postcardiotomy mechanical support is, therefore, rarely required. Extracorporeal membrane oxygenation (ECMO) constitutes an effective and essentially the only available short-term circulatory support for acute postoperative severe cardiopulmonary failure in the paediatric population following cardiac surgery. Nonetheless, it has been associated with increased risk of complications including bleeding, thromboembolism, plasma leakage, and infection [6, 7].

## 2. Case Report

### 2.1. Case I

An 8-month-old boy with Williams' syndrome and supravalvular aortic stenosis was admitted to our department for surgical treatment. Repair was achieved by extended aortoplasty with incision of noncoronary and right coronary sinuses and implantation of an inverted Y-shaped Dacron patch. Failure to wean from cardiopulmonary bypass (CPB) due to postcardiotomy cardiac failure resulted in the institution of extracorporeal membrane oxygenation (ECMO). Reexploration for bleeding was required the first day. The patient was successfully weaned off ECMO support 70 hours later. ICU and total hospital stay was 9 and 16 days, respectively. The patient is alive at 5-year follow-up with excellent cardiac function.

### 2.2. Case II

A 13-month-old girl with the Williams-Beuren syndrome and supravalvular aortic and pulmonary stenosis was admitted to our department for surgical treatment. Repair technique included patch enlargement, extended aortoplasty with incision of the noncoronary and right coronary sinus, and implantation of an inverted Y-shaped Dacron patch. Inability to wean from CPB due to postcardiotomy cardiac failure led to the institution of ECMO for 44 hours. The patient remained in ICU stay for 30 days and the hospital for a total of 39 days. In the postoperative course she developed pneumothorax after the removal of the chest drain tubes and was treated accordingly. The patient is alive at 5-year follow-up with excellent cardiac function.

The ECMO system, in both cases, was instituted utilizing the original CPB cannulae. The circuit included a Levitronix CentriMag (Levitronix LLC, Waltham, MA, USA) centrifugal pump, a hollow fibre membrane gas exchanger, and heparin-bonded tubing. Conservative systemic heparinization was used until mediastinal bleeding was well controlled; adequate anticoagulation was thereafter achieved by heparin sodium administration at a dose of 20–40 u/kg/hr in order to maintain an activated clotting time (ACT) of 140–180 sec. Mean ECMO flow was maintained at 150 mL/kg/min (range 60–250 mL/kg/min) depending on cardiac function, serum lactate levels, and mixed venous oxygen saturation. Minimal inotropic maintenance was continued during mechanical support (adrenaline 0.05–0.08 mcg/kg/min, dopamine 5–8 mcg/kg/min, and milrinone 0.25–0.50 mcg/kg/min) to facilitate left ventricular (LV) emptying against increased afterload and prevent overdistension. Ventilation (25–50% of calculated settings) was maintained to keep the lungs inflated and avoid alveolar collapse. Weaning from ECMO was accomplished by maximising inotropic support and gradually decreasing flow rates similarly to weaning from CPB. Once the patient was completely off support, haemodynamic stability was monitored and tissue perfusion was assessed by arterial blood gases with serum lactate and base deficit values. Cannulae were subsequently removed. Both patients made a full recovery and were discharged from hospital in excellent clinical condition (Table 1). Five years later they remain well with excellent cardiac function.

## 3. Discussion

Localization of the stenosis at the level of the commissures of the aortic valve in patients with SVAS has important implications for both aortic valve function and the coronary circulation. Coronary orifices can be obstructed by the thickened aortic or sinus wall and these patients should be considered at risk of myocardial ischemia. Furthermore, the coronary arteries are subjected to the elevated prestenotic systolic pressure, resulting in dilatation and tortuosity [4, 5]. Sudden death during certain procedures, attributed to acute myocardial ischemia, has been reported associated with a sudden drop in coronary perfusion pressure [8]. Although our patients had not undergone coronary arteriography and therefore coronary artery obstruction cannot be demonstrated, it is, however, reasonable to assume that coronary hypoperfusion would be responsible for poor myocardial preservation. Nonetheless, it is also true that patients with severe pulmonary artery stenosis present with significant right ventricular pressure overload and therefore bear a higher operative risk [7].

Extracorporeal membrane oxygenation for circulatory support is established via venoarterial and, in the case of postoperative cardiac failure, intrathoracic and central (right atrium and aorta) routes. A suitable level of anticoagulation has to be maintained, which increases the risk of bleeding. Other reported complications include thromboembolism, haemolysis, renal failure, sepsis, or vascular complications [6, 7, 9]. The Levitronix CentriMag device is a centrifugal pump that consists of a magnetically levitated bearing-less rotor designed to reduce blood friction. It operates with minimal shear trauma to blood cells, reducing the risks of haemolysis and thrombus formation. The risk of thrombus formation is reduced by uniform unidirectional flow and less stagnation, while reduced shearing stress attenuates haemolysis [7].

There was no evidence of clots in the pump head, tubing, or cannulae after their removal in our patients. The other markers of blood trauma including serum bilirubin and lactate dehydrogenase (LDH) remained within the normal range. The devise was not demanding to handle and easy to monitor. The Levitronix CentriMag device provided successful mechanical support in our young patients with acute postcardiotomy heart failure with minimal anticoagulation. Larger studies are required to further elucidate the efficacy of this device and help draw guidelines regarding anticoagulation.

Although it is difficult to draw firm conclusions from just 2 patients, yet in conjunction with other reported cases, SVAS patients should receive special attention to their coronary circulation, which in our cases was probably responsible for poor myocardial preservation during cardiac surgery.

## Figures and Tables

**Table 1:** Cases summary.

	Case 1	Case 2
Age (months)	8.5	13
Gender	Male	Female
Weight	6.7 kg	9 kg
Height	71 cm	70 cm
Primary diagnosis	Supravalvular aortic stenosis, Williams' syndrome	Supravalvular aortic stenosis, pulmonary artery stenosis, Williams-Beuren syndrome
CPB time	226 min	159 min
Ischaemic time	61 min	54 min
ECMO support	70 hrs	44 hrs
APPT (for ACT 140–180)	48.9* (32.3–>110)	35.2* (31.4–89.4)
Complications	Haemorrhage and reexploration	Pneumothorax
ICU stay	9 days	30 days
Total stay	16 days	39 days

*Median value.

## Abbreviations

SVAS: Supravalvar aortic stenosis
ECMO: Extracorporeal membrane oxygenation
CPB: Cardiopulmonary bypass
ACT: Activated clotting time.
